# Brain parenchymal and leptomeningeal metastasis in non-small cell lung cancer

**DOI:** 10.1038/s41598-022-26131-z

**Published:** 2022-12-26

**Authors:** Qing Li, Zhen Lin, Ye Hong, Yang Fu, Yueyun Chen, Ting Liu, Yue Zheng, Jiangfang Tian, Chunhua Liu, Wei Pu, Zhenyu Ding, Chun Wang

**Affiliations:** 1 Department of Biotherapy, Cancer Center, West China Hospital, Sichuan University, Chengdu, Sichuan China; 2 Division of Internal Medicine, Department of Endocrinology and Metabolism, West China Hospital, Sichuan University, No. 37 Guo Xue Alley, Chengdu, 610041 Sichuan China

**Keywords:** Cancer, Neurology, Risk factors

## Abstract

Patients with advanced non-small cell lung cancer (NSCLC) are prone to brain metastases (BM), which essentially include brain parenchymal metastases (PM) and leptomeningeal metastases (LM). We conducted a retrospective study to comprehensively assess the clinical characteristics and risk factors of patients with advanced NSCLC who develop PM and LM. Patients with advanced NSCLC were enrolled. These patients were then divided into three groups for analysis: patients without BM (No-BM), patients with PM and patients with LM. Data on clinical characteristics of each patient at the time of diagnosis advanced NSCLC were extracted and analyzed. In addition, prediction models were developed and evaluated for PM and LM. A total of 592 patients were enrolled in the study. BM was present in 287 patients (48.5%). Among them, 185 and 102 patients had PM or LM. Patients with LM had a higher proportion of EGFR exon 21point mutations (L858R) compared to patients with No-BM and PM (p < 0.0001). The median time to the onset of PM and LM from the diagnosis of advanced NSCLC was 0 months and 8.3 months, respectively. Patients with LM had a statistically shorter over survival (OS) compared to either No-BM or PM patients (p < 0.0001). Based on independent predictive variables, two nomogram models were constructed to predict the development of PM and LM in advanced NSCLC patients, and the C-indexes were 0.656 and 0.767, respectively. Although both considered as BM, PM and LM had different clinical characteristics. And the nomogram showed good performance in predicting LM development, but not PM.

## Introduction

Lung cancer is the leading cause of cancer-related deaths worldwide and poses a serious threat to human health and life^1^. Because of its insidious onset, most patients are already at an advanced stage by the time they are diagnosed. As non-small cell lung cancer (NSCLC) accounts for the vast majority of lung cancers and the propensity of NSCLC to spread to the brain^2, 3^, brain metastases (BM), a substantial contributor to overall cancer mortality, commonly arises in patients with advanced NSCLC^4, 5^. Given the associated neurological symptoms, psychological impact, changes in tumor treatment plans, and restrictions on eligibility for clinical trials, the development of BM can significantly alter the clinical course of patients and pose distinct clinical challenges^6^.

Research on BM can identify new treatment targets and determine new therapeutic approaches to address unmet clinical needs. However, the paucity of preclinical models of BM has severely limited the progress of research on BM^4^. The patient's baseline characteristics, tumor metastasis sites, and tumor markers are closely related to tumor occurrence, evolution and prognosis. Exploring the clinical features of BM and identifying risk factors involved in BM occurrence from a clinical perspective may lead to a more accurate diagnosis and clinical management, as well as the design of more effective treatments^7^.

It is worth noting that the brain actually comprises two main microenvironments, the brain parenchyma and the leptomeninges, which differ in cell types, anatomical structures, metabolic constraints and immune environment. This imposes a distinct and profound selective pressure on tumour cells that, in turn, shapes the clinical characteristics and therapeutic responses^8^. However, few, if any, studies have been reported on whether their respective clinical characteristics, survival prognosis, and risk factors for occurrence are different^9–25^. Therefore, we conducted this study to comprehensively assess the clinical characteristics, survival prognosis, and risk factors of patients with advanced NSCLC who develop brain parenchymal metastases (PM) and leptomeningeal metastasis (LM).

## Methods

### Study design and patients

The retrospective, observational study was conducted in West China Hospital and was performed in accordance with the Declaration of Helsinki. The ethical committee of the hospital reviewed and approved the study protocol (2021-1349), and subjects were exempted from informed consent. All the authors attest that the study was conducted in accordance with the protocol and all its amendments. All the authors had access to the data used for the writing of the manuscript and vouch for the accuracy and completeness of the data and analyses.

**Figure 1:**
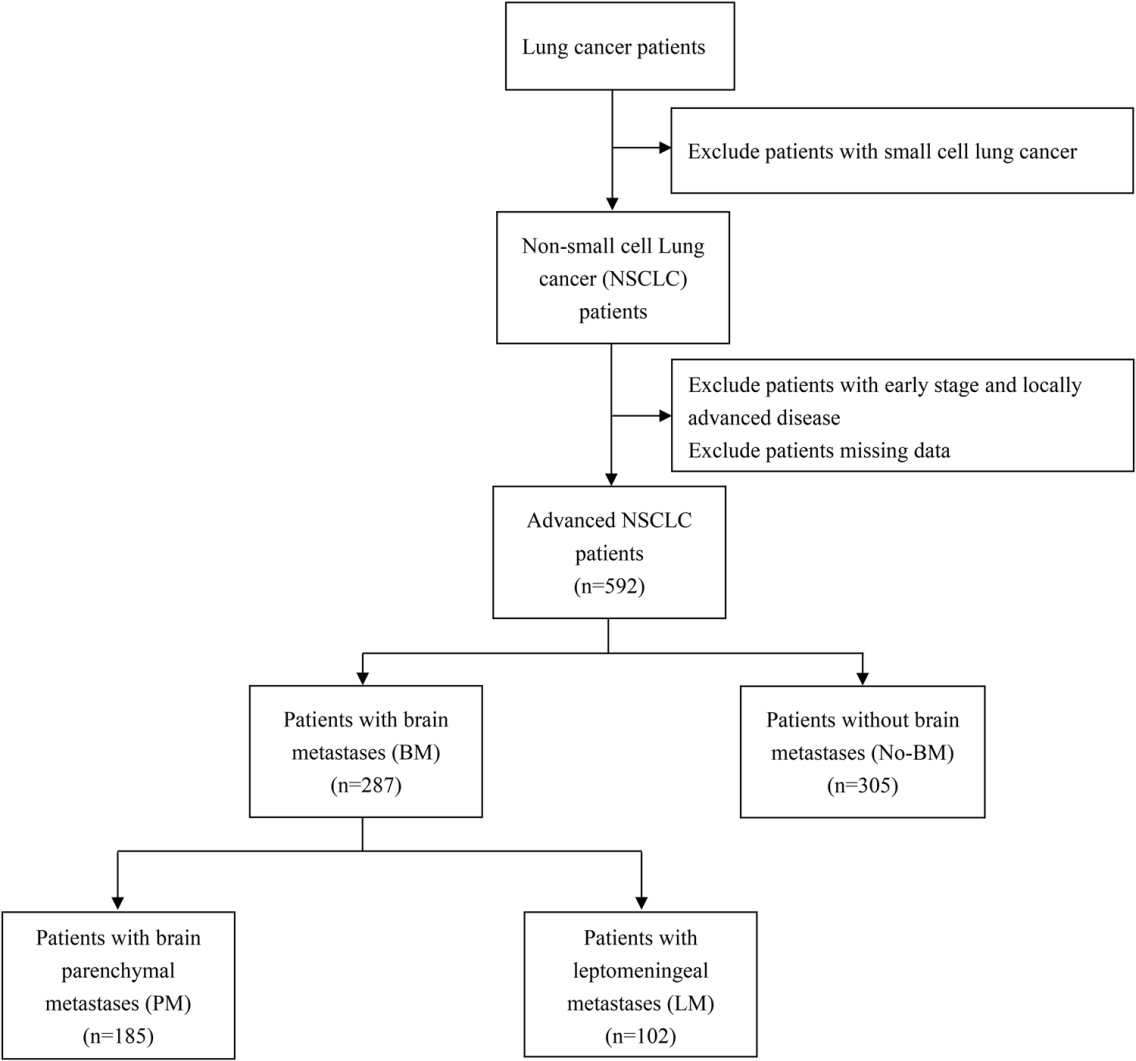
Study flowchart.

Patients were screened through the Hospital Information System (HIS) electronic files from January 2010 to October 2021. Individuals who had pathologically confirmed lung cancer were screened. Patients were excluded if they were diagnosed with small cell lung cancer (SCLC); they were documented as having early stage or locally advanced disease; or they were missing data on the timing of their diagnosis, tumor stage, or histology. These advanced NSCLC patients were then divided into 2 groups for analysis: patients with BM and patients without BM (No-BM). Among them, patients with BM were further divided into patients with PM, or LM. The study flowchart was shown in Fig. 1. In this study, PM was diagnosed by magnetic resonance imaging (MRI) screening. The diagnosis of LM was dependent on MRI and/or positive cytology findings in cerebrospinal fluid (CSF). Pure symptoms, whether at initial consultation or during follow-up, were not accepted. The patients not met the diagnostic criteria were defined as No-BM.

### Data collection

Retrieved data regarding demographic features, date of advanced NSCLC and BM diagnosis, histology, EGFR mutation status and clinical outcomes. We collected the demographic features, metastatic sites and tumor markers at the time of diagnosis of advanced NSCLC. Tumor markers mainly included carcinoembryonic antigen (CEA), cytokeratin 19 (CYFRA21-1), and neuron-specific enolase (NSE).

### Nomogram model

Binary logistic regressions were employed to determine the significant characteristics with the help of the backward stepwise selection method. The Harrell’s concordance index (C-index), Brier score and the receiver operating characteristic (ROC) were used to estimate the predictive performance of the nomogram. Internal validation of the nomogram was performed by using the Hosmer–Lemeshow test, bootstrapping with 1000 samples and tenfold cross-validation method. In addition, decision curve analysis (DCA), which plots the net benefit (NB) within a reasonable risk threshold consistent with clinical practice, was applied to assess the clinical utility of nomograms in decision making. Based on DCA, we developed clinical impact plots that visualize the number of estimated high-risk patients for each risk threshold.

### Statistics

Continuous variables were presented as median values (min–max), and categorical variables were summarized as proportions. Continuous variables and categorical variables were compared by the Kruskal–Wallis test and Chi-squared test, respectively. The cut-off values of the continuous variables were based on median value, except for age. The median overall survival was estimated by using the Kaplan–Meier method. IBM SPSS Statistics, version 26.0 (SPSS Inc, Chicago, IL, USA) and R software version 4.1.2 (http://www.r-project.org) performed the statistical methods mentioned above, and several R packages, including regplot, rms, rmda, survival, and pROC were applied to draw graphs, such as nomogram, calibration plot, DCA plot, and ROC curve. All p values were two-sided with values of p < 0.05 were considered statistically significant, and confidence intervals (CIs) stated at the 95% confidence level.

## Results

### Baseline characteristics

A total of 592 patients were included. BM was present in 287 patients (48.5%). Among the patients with BM, 185 (64.5%) patients had PM, and 102 (35.5%) patients had LM (Fig. 1). The demographic and clinical characteristics of these patients at the time of diagnosis of advanced lung cancer were shown in Table 1. For the overall cohort, the median age was 58.5 years (range 21–85), and the majority of patients were female (59.6%), non-smokers (74.2%), and had adenocarcinoma (96.3%). EGFR mutation was the most frequent driver mutation (85.0%). Among these patients, the most common site of tumor metastasis was lymph node (63.2%), followed by lung (54.4%) and then bone (44.8%). Most patients were free of pleural (64.0%), liver (90%), and adrenal metastases (91.7%). The median values of tumor markers CEA, CYFRA21-1, and NSE were 14.29 (range 0.45–1000.00), 3.96 (range 0.80–500.00), and 14.28 (range 2.69–1494.00), respectively.

**Table 1:** Demographic and clinical characteristics of patients included in the analysis.

Characteristics	Overall cohort (n = 592)	No-BM (n = 305)	PM (n = 185)	LM (n = 102)
**Sex, n (%)**				
Male	239 (40.4%)	127 (41.6%)	72 (38.9%)	40 (39.2%)
Female	353 (59.6%)	178 (58.4%)	113 (61.1%)	62 (60.8%)
Age, median (min–max)	58.50 (21–85)	61 (21–83)	57 (30–79)	55 (30–85)
**Smoking, n (%)**				
Yes	153 (25.8%)	78 (25.6%)	47 (25.4%)	28 (27.5%)
No	439 (74.2%)	227 (74.4%)	138 (74.6%)	74 (72.5%)
**ECOG PS, n (%)**				
< 2	546 (92.2%)	296 (97.0%)	176 (95.1%)	74 (72.5%)
≥ 2	46 (7.8%)	9 (3.0%)	9 (4.9%)	28 (27.5%)
**Histology, n (%)**				
Adenocarcinoma	570 (96.3%)	293 (96.1%)	179 (96.8%)	98 (96.1%)
Non-adenocarcinoma	22 (3.7%)	12 (3.9%)	6 (3.2%)	4 (3.9%)
**EGFR mutations, n (%)**				
19Del	259 (43.8%)	149 (48.9%)	86 (46.5%)	24 (23.5%)
L858R	244 (41.2%)	113 (37.0%)	77 (41.6%)	54 (53.0%)
Others (unknown or no)	89 (15.0%)	43 (14.1%)	22 (11.9%)	24 (23.5%)
**Lymph node metastasis, n (%)**				
Yes	374 (63.2%)	181 (59.3%)	129 (69.7%)	64 (62.7%)
No	218 (36.8%)	124 (40.7%)	56 (30.3%)	38 (37.3%)
**Liver metastasis, n (%)**				
Yes	59 (10.0%)	20 (6.6%)	25 (13.5%)	14 (13.7%)
No	533 (90.0%)	285 (93.4%)	160 (86.5%)	88 (86.3%)
**Lung metastasis, n (%)**				
Yes	322 (54.4%)	165 (54.1%)	97 (52.4%)	60 (58.8%)
No	270 (45.6%)	140 (45.9%)	88 (47.6%)	42 (41.2%)
**Bone metastasis, n (%)**				
Yes	265 (44.8%)	123 (40.3%)	86 (46.5%)	56 (54.9%)
No	327 (55.2%)	182 (59.7%)	99 (53.5%)	46 (45.1%)
**Pleural metastasis, n (%)**				
Yes	213 (36.0%)	136 (44.6%)	54 (29.2%)	23 (22.5%)
No	379 (64%)	169 (55.4%)	131(70.8%)	79 (77.5%)
**Adrenal metastasis, n (%)**				
Yes	49 (8.3%)	11 (3.6%)	21 (11.4%)	17 (16.7%)
No	543 (91.7%)	294 (96.4%)	164 (88.6%)	85 (83.3%)
CEA (ng/ml), median (min–max)	14.29 (0.45–1000.00)	10.03 (0.66–1000.00)	21.72 (0.45–1000.00)	21.59 (0.45–1000.00)
CYFRA21-1 (ng/ml), median (min–max)	3.96 (0.80–500.00)	4.05 (0.92–146.40)	4.35 (0.82–139.60)	3.45 (0.80–500.00)
NSE (ng/ml), median (min–max)	14.28 (2.69–1494.00)	13.65 (2.88–1494.00)	14.91 (2.69–422.99)	14.41 (7.63–60.25)

**Figure 2:**
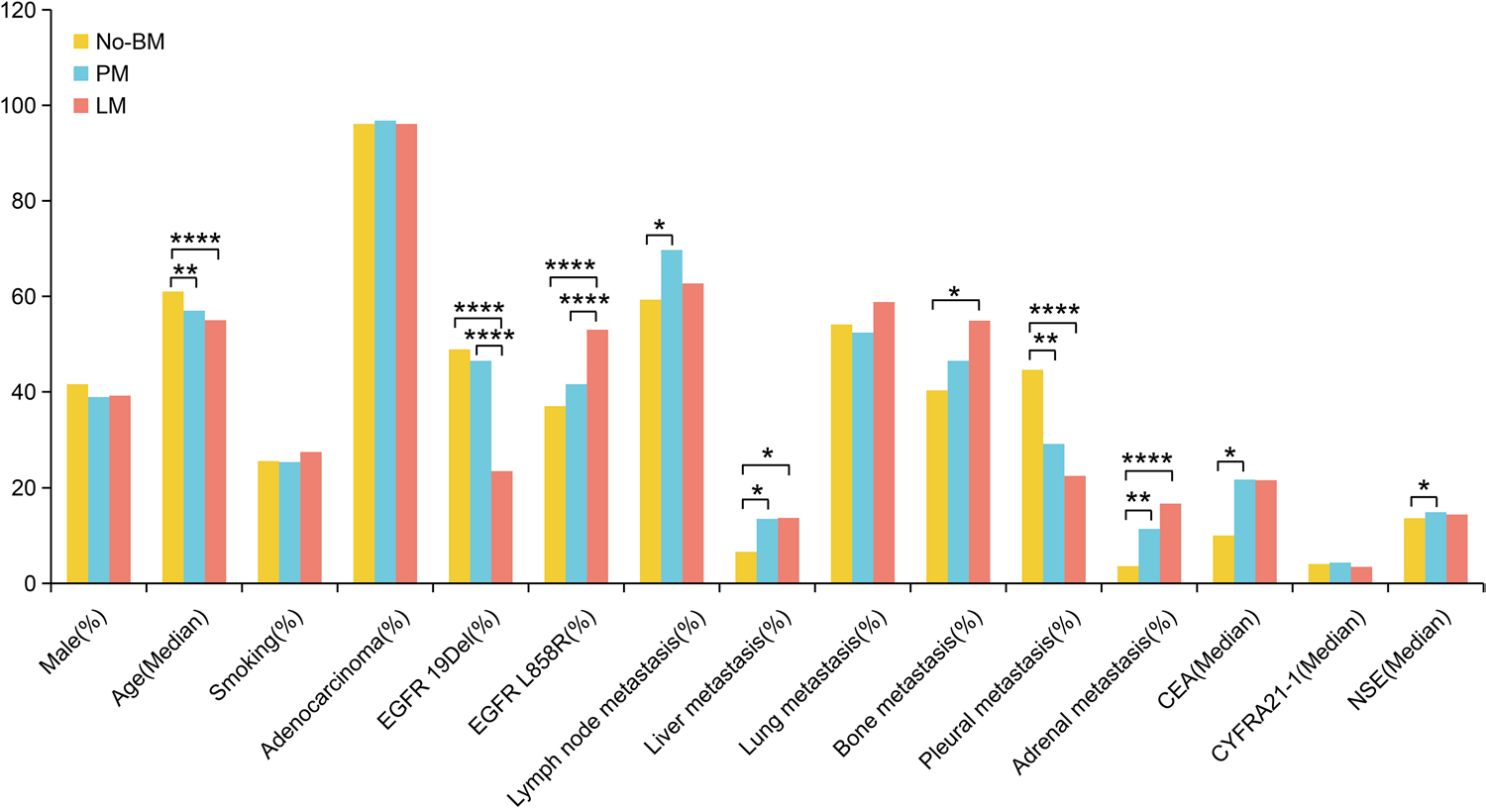
Baseline characteristics. *p < 0.05, **p < 0.01, ****p < 0.0001.

We further compared the baseline characteristics of patients in each group, and we found no differences among these groups in terms of sex, smoking history, type of histology, proportion of lung metastases and CYFRA21 levels at the time of diagnosis of advanced NSCLC (Fig. 2). Patients who developed PM and LM were younger compared to No-BM patients (p < 0.05). Patients with LM had a lower proportion of EGFR exon 19 deletion mutations and a higher proportion of exon 21 point mutations (L858R) or other types of mutations compared to patients with No-BM and PM (p < 0.0001). There were more patients with lymph node metastasis in PM patients (p = 0.021). And patients who developed PM and LM had a higher proportion of liver metastases and adrenal metastasis, but a lower proportion of pleural metastases, compared with No-BM patients (p < 0.05). A higher proportion of bone metastases occurred in the LM group (p = 0.010).

### Time of PM and LM onset

**Figure 3:**
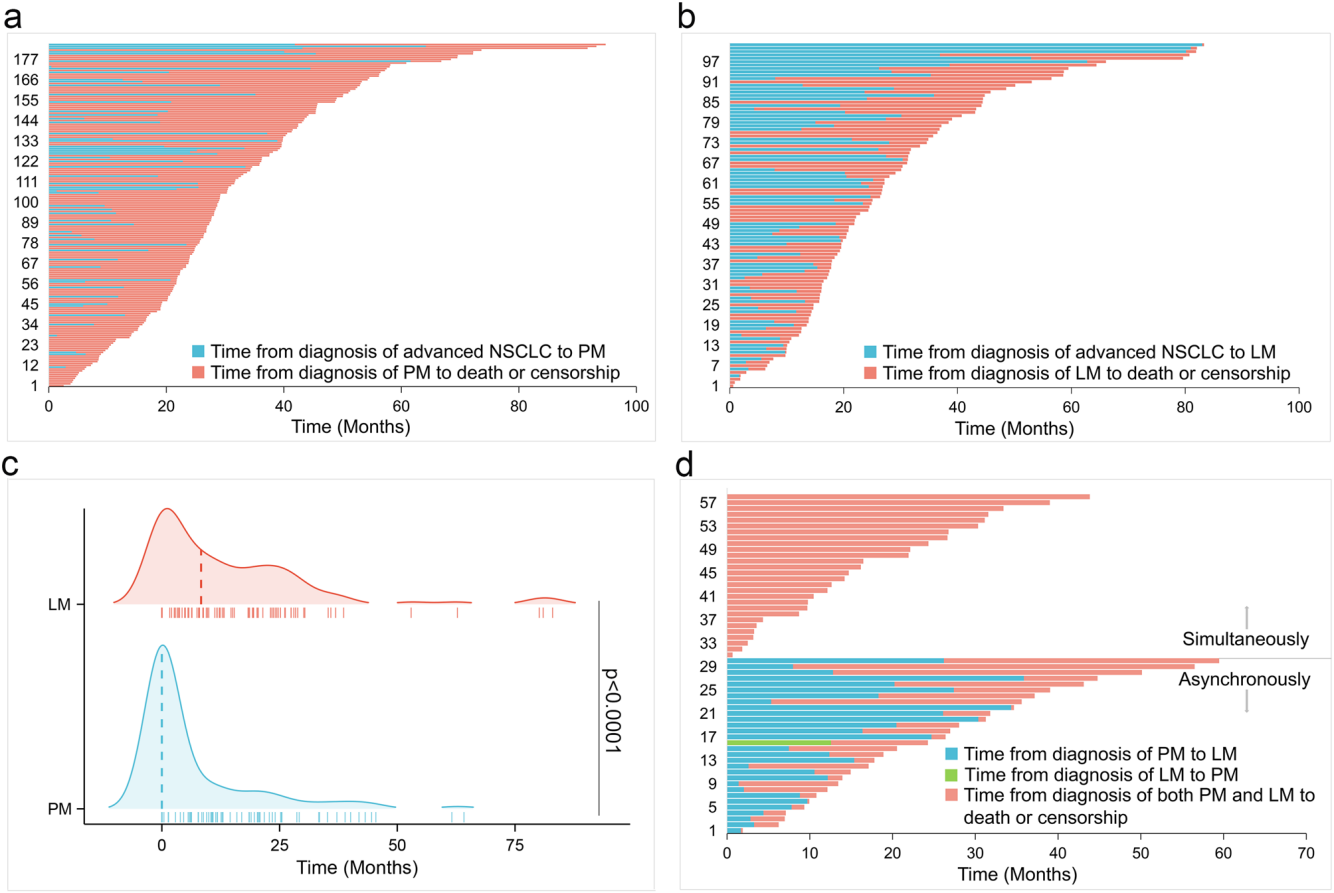
The time of PM and LM onset. The swimming plot from the diagnosis of advanced NSCLC to the onset of PM (**a**) or LM (**b**) and eventually to death or censorship. Summary of the onset of PM or LM during the clinical course of advanced NSCLC (**c**). The swimming plot of the patients with both LM and PM (**d**).

Of the patients with PM (n = 185), the majority (68.1%) had this co-morbidity at baseline (Fig. 3a). However, in patients with LM (n = 102), only 32.4% patients were simultaneously diagnosed of LM and advanced NSCLC, while the most had LM onset later during the treatment course (Fig. 3b). The median time to the establishment of PM or LM from the first sign of metastases was 0 months (range 0–64.1 months) and 8.3 months (range 0–83.0 months), respectively (Fig. 3c). Notably, 59 patients in LM cohort had both LM and PM. These patients either had concurrent LM and PM (n = 29) or sequential LM after PM (n = 29). Only 1 patient developed PM after LM (Fig. 3d).

### Metastatic sites

**Figure 4:**
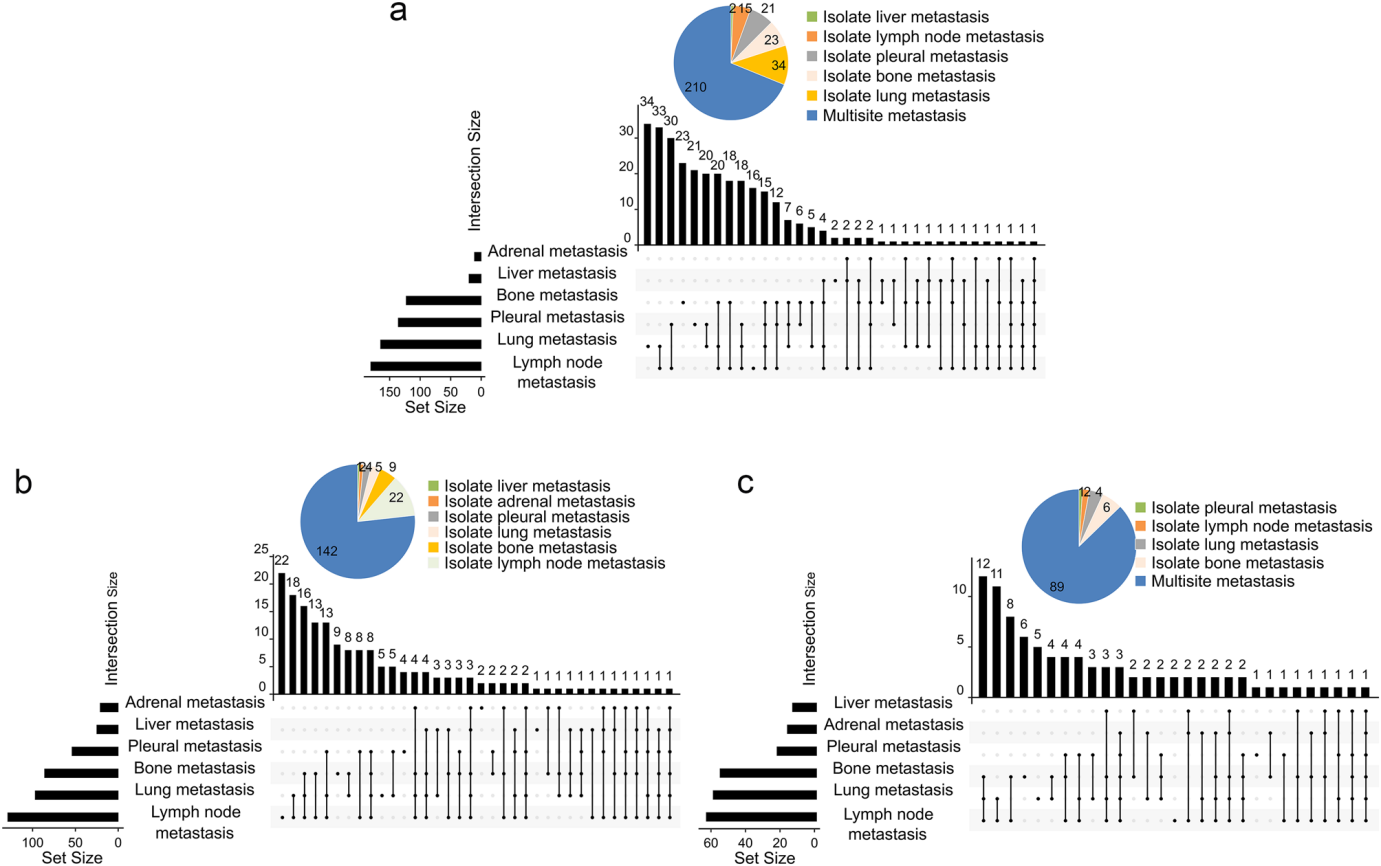
Metastatic sites. The summary of metastatic sites in patients with No-BM (**a**), PM (**b**) and LM (**c**).

Our patients were prone to multiple metastases (≥ 2 metastatic sites) (Fig. 4). The proportions of No-BM, PM and LM with multiple metastases were 68.9%, 76.8% and 87.3%, respectively (Fig. 4). And multiple metastases were significantly increased in LM patients relative to No-BM (p < 0.0001) and PM patients (p = 0.010). For the patients with No-BM, the most common metastatic site was lymph node, followed by the lung, pleural, bone, liver, and adrenal gland (Fig. 4a). For the patients with PM, the ranking of the metastatic sites was lymph node, lung, bone, pleural, liver, and adrenal gland (Fig. 4b). For LM patients, the ranking of the common metastatic sites was similar to that of PM patients, with the exception of adrenal gland and liver metastases (Fig. 4c).

### Survival

**Figure 5:**
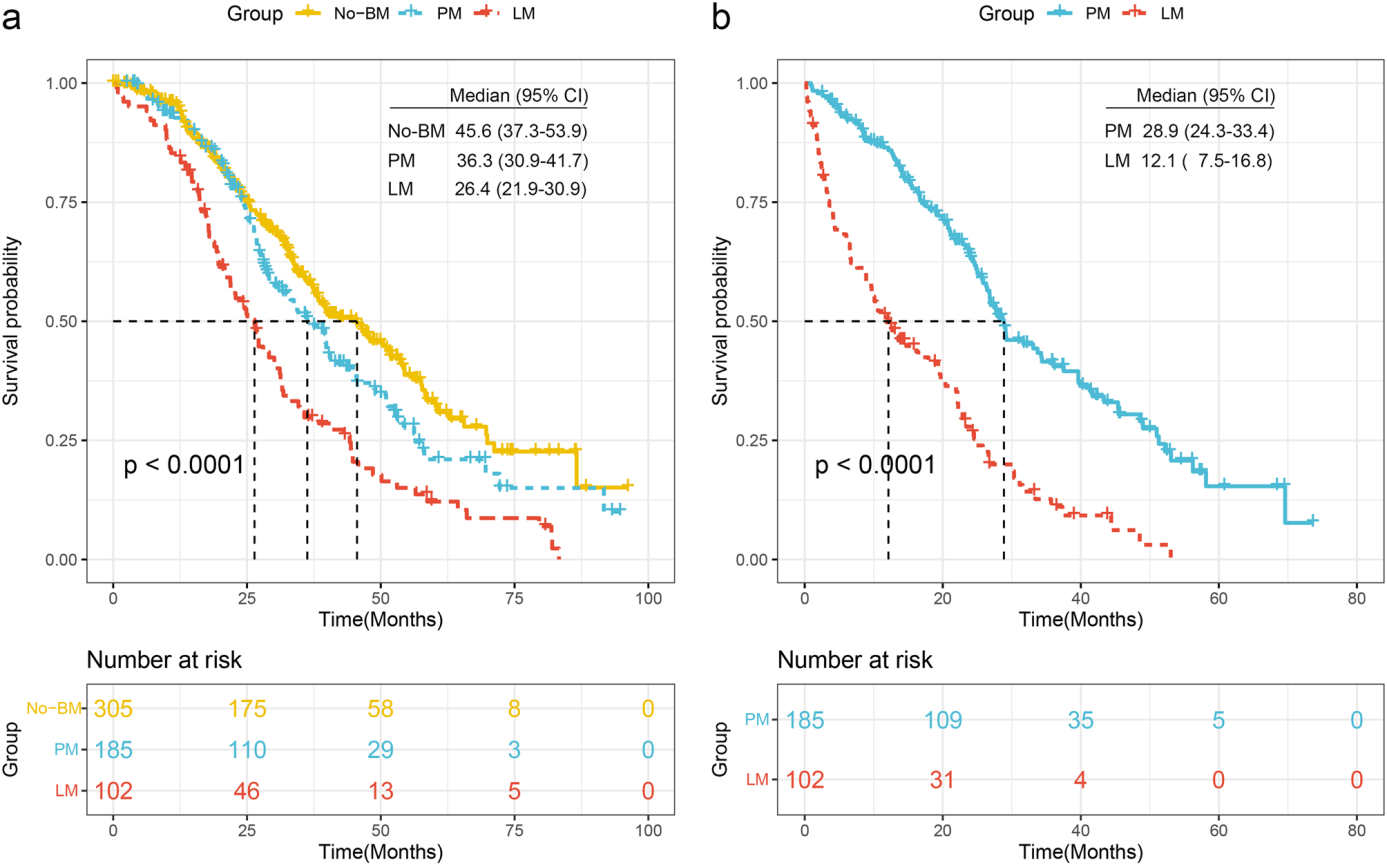
Survival. The survival curves calculated from the diagnosis of advanced NSCLC (**a**). The survival curves calculated from the diagnosis of PM and LM (**b**).

The median overall survival (OS) of the whole cohort was 36.9 months (95% CI 33.8–39.9 months), from the onset of metastasis. Compared with those with No-BM (45.6 months, 95% CI 37.3–53.9 months), patients with PM (36.3 months, 95% CI 30.9–41.7 months, p = 0.056) and LM (26.4 months, 95% CI 21.9–30.9 months, p < 0.0001) had poor survival (Fig. 5a). Besides, those with LM even had a worse prognosis than their counterparts with PM (p < 0.0001) (Fig. 5a). When calculated from the onset of BM, the patients with PM (28.9 months, 95% CI 24.3–33.4 months) still live favorably over those with LM (12.1 months, 95% CI 7.5–16.8 months, p < 0.0001, Fig. 5b).

### Risk factors

The age, lymph node, liver, pleural, adrenal metastasis, CEA and NSE were the risk factors for PM (Table 2). In multivariable analysis, age, pleural metastasis, adrenal metastasis, CEA and NSE were independent variables associated with PM (Table 2). For LM, age, EGFR mutations, liver, bone, pleural, adrenal metastasis and CEA were risk factors, and age, EGFR mutation, pleural metastasis, adrenal metastasis and CEA remained in the multivariate analysis (Table 2).

**Table 2:** Univariate and multivariate logistic analysis of the variables associated with the PM and LM.

Characteristics	No-PM vs PM				No-BM vs LM			
	Univariable OR (95% CI)	p value	Multivariable OR (95% CI)	p value	Univariable OR (95% CI)	p value	Multivariable OR (95% CI)	p value
Sex (male vs female)	1.121 (0.779–1.614)	0.539			1.106 (0.699–1.748)	0.667		
Age (< 60 vs ≥ 60)	0.667 (0.464–0.960)	0.029	0.692 (0.459–1.043)	0.078	0.376 (0.230–0.613)	< 0.0001	0.318 (0.181–0.559)	< 0.0001
Smoking (yes vs no)	0.991 (0.658–1.492)	0.966			1.101 (0.664–1.825)	0.708		
Histology (ade vs non-ade)	0.800 (0.302–2.117)	0.653			0.997 (0.314–3.162)	0.995		
**EGFR mutations**		0.462				< 0.0001		< 0.0001
19Del	(reference)				(reference)		(reference)	
L858R	1.048 (0.714–1.538)	0.811			2.967 (1.730–5.088)	< 0.0001	2.641 (1.441 -4.841)	0.002
Others (unknown or no)	0.735 (0.420–1.286)	0.281			3.465 (1.791–6.702)	< 0.0001	4.087 (1.934–8.636)	< 0.0001
Lymph node metastasis (yes vs no)	1.607 (1.099–2.348)	0.014			1.154 (0.727–1.831)	0.544		
Liver metastasis (yes vs no)	2.019 (1.124–3.627)	0.019			2.267 (1.100–4.674)	0.027		
Lung metastasis (yes vs no)	0.906 (0.634–1.295)	0.589			1.212 (0.770–1.909)	0.406		
Bone metastasis (yes vs no)	1.188 (0.830–1.700)	0.347			1.801 (1.146–2.831)	0.011		
Pleural metastasis (yes vs no)	0.570 (0.389–0.835)	0.004	0.495 (0.324–0.756)	0.001	0.362 (0.216–0.606)	< 0.0001	0.307 (0.172–0.547)	< 0.0001
Adrenal metastasis (yes vs no)	2.493 (1.281–4.854)	0.007	1.922 (0.944–3.914)	0.072	5.345(2.412–11.848)	< 0.0001	4.418 (1.811–10.777)	0.001
CEA (< 14.29 vs ≥ 14.29 ng/ml)	1.772 (1.121–2.595)	0.003	1.802 (1.204–2.698)	0.004	1.750 (1.092–2.802)	0.020	1.780 (1.052–3.013)	0.032
CYFRA21-1 (< 3.96 vs ≥ 3.96 ng/ml)	1.149 (0.783–1.685)	0.477			0.635 (0.392–1.027)	0.064		
NSE (< 14.28 vs ≥ 14.28 ng/ml)	1.594 (1.081–2.350)	0.019	1.504 (1.005–2.251)	0.047	1.361 (0.834–2.221)	0.217		

### Predictive models

**Figure 6:**
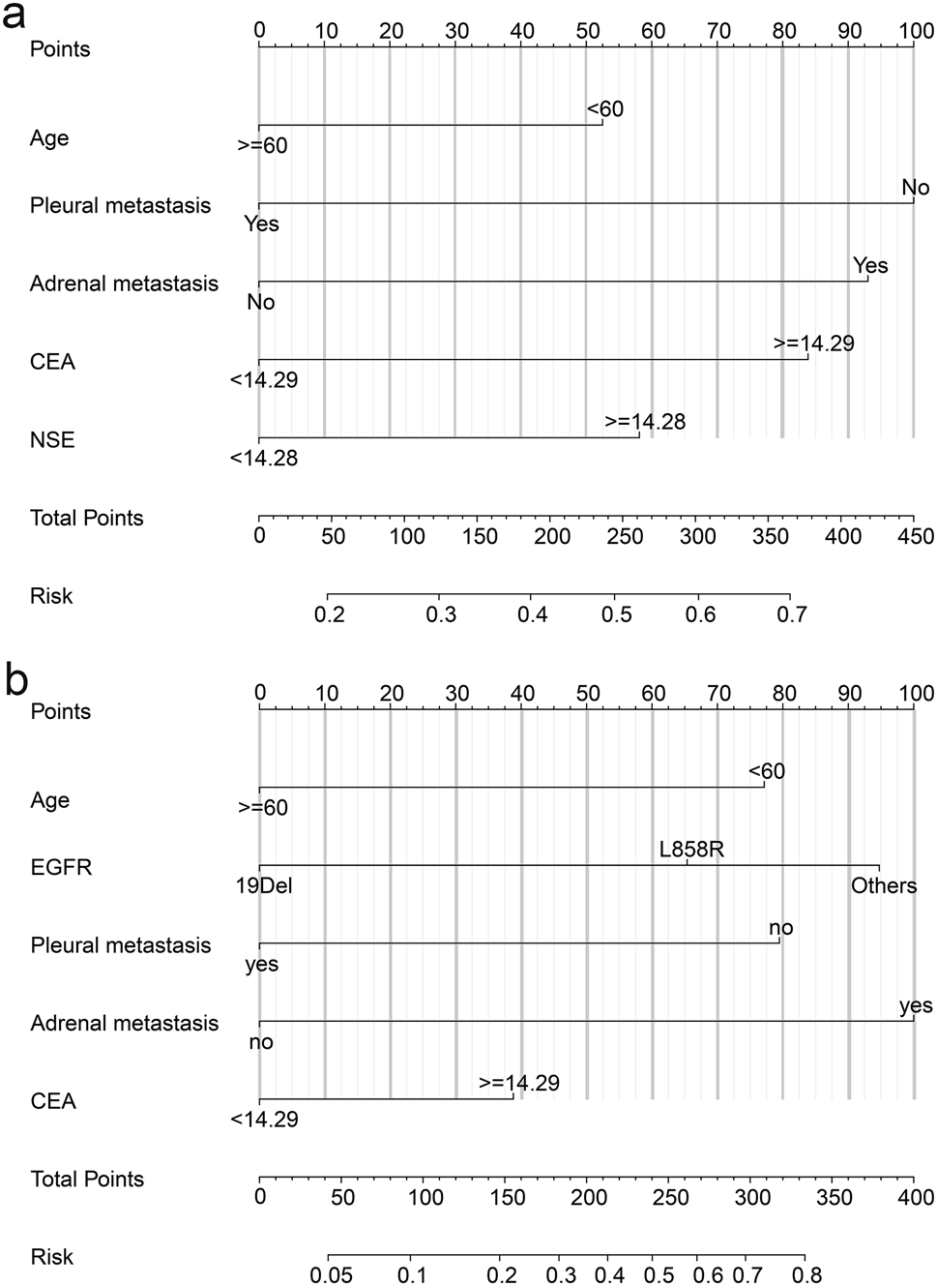
Nomogram for the prediction of PM (**a**) and LM (**b**).

**Figure 7:**
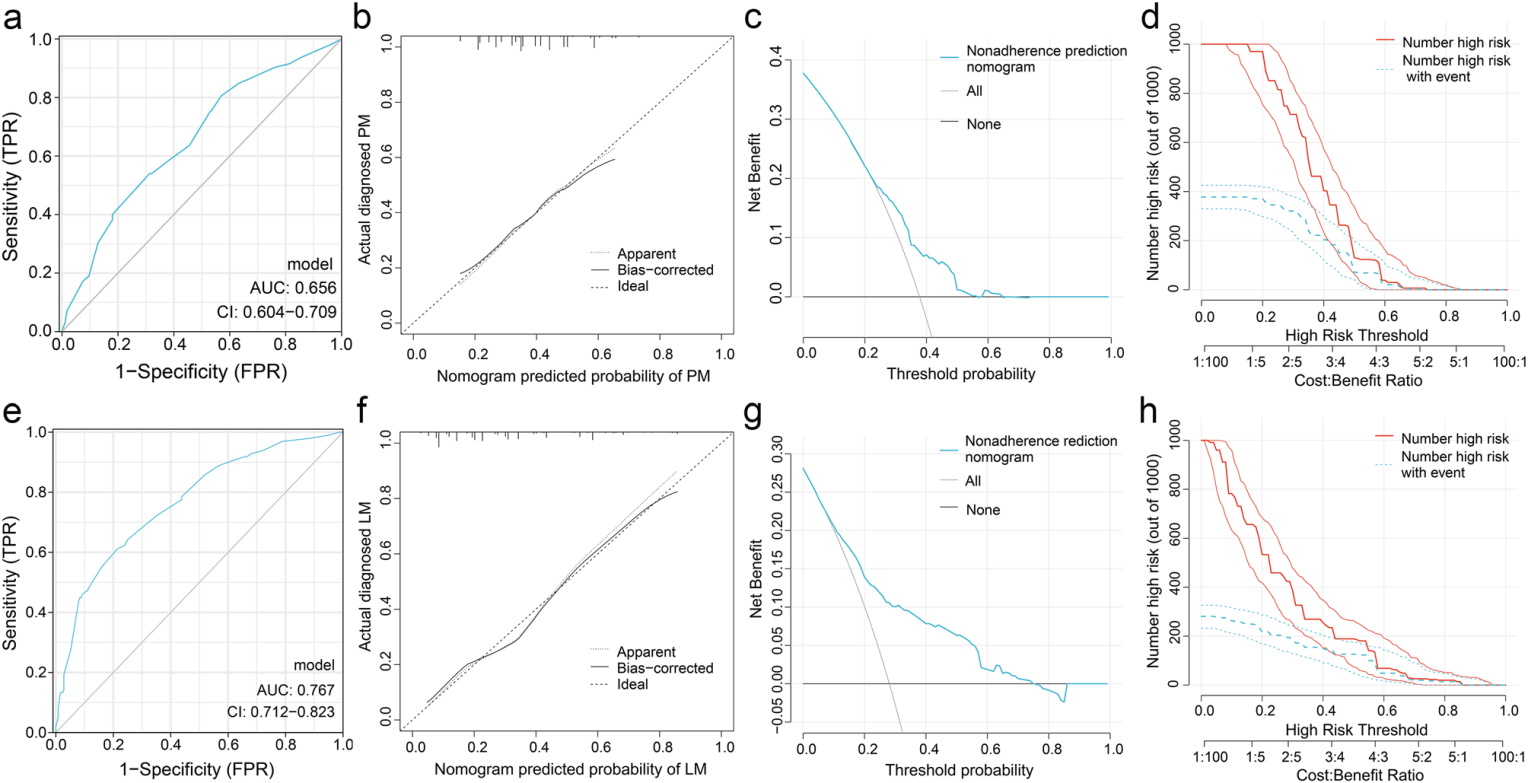
Receiver operating characteristic (ROC) curves (**a**,**e**), calibration curves (**b**,**f**), decision curve analysis (DCA, **c**,**g**) and clinical impact plots of nomogram (**d**,**h**) for predicting PM and LM.

Based on previous results, the PM and LM nomogram models were constructed, respectively (Fig. 6). The C-indexes in the PM and LM groups were 0.656 (95% CI 0.604–0.709) and 0.767 (95% CI 0.712–0.823), respectively. And the Brier score was 0.219 and 0.162 in the PM and LM groups. By tenfold cross-validation, the accuracy of the PM group was 63.4%, while the accuracy of the LM group was 78.0%. Meanwhile, in terms of ROC curves (Fig. 7a,e), calibration curves (Fig. 7b,f), DCA cures (Fig. 7c,g) and clinical impact plots (Fig. 7d,h), these nomograms seemed to be as an excellent predicting tool for LM in newly diagnosed advanced NSCLC patients but not for PM.

## Discussion

In this study, the clinical characteristics of patients with PM or LM were compared. We found patients who developed PM and LM had different characteristics, such as EGFR mutations, time of PM or LM onset, proportion of multiple metastases, and survival. These results suggested that although both PM and LM were considered BM, they were different in the biological behavior and clinical course.

We found PM or LM occurrence was not a random event across lung cancer patients, but to some extent enriched in the patients with distinct feature. For instance, young patients were more susceptible to PM or LM. It should be noted young patients with lung cancer were more vulnerable to driver mutation. Patients harboring EGFR L858R were more likely to develop LM than those harboring EGFR 19Del, reported by previous studies as well^21^. Patients with PM or LM were prone to multiple metastases (Fig. 4), and liver and adrenal metastases (Fig. 2). These clinical features were particularly significant in patients with LM. And LM patients had a higher proportion of bone metastases. The mechanisms were unknown right now, but it might be a clinical manifestation of the genetic aberrations in different tumors.

Our results showed that LM was a later event compared to PM in advanced NSCLC (Fig. 3a–c). The leptomeninges contain circulating CSF, which is significantly hypoxic and has low concentrations of metabolic intermediates and micronutrients. Cancer cells must "overcome" these selection pressures and become more aggressive in sequence. This may partially explain the more advanced appearance of LM.

In addition, in our study, more than half of the patients with LM also had PM (Fig. 3d). In these patients, LM appeared either with or following PM, with the exception of only 1 patient (Fig. 3d). This result was consistent with our previous work, indicating that PM might be a risk factor for LM^18^. Although the mechanism beneath remained elusive, we reasonably inferred cancer cells evolved to acquire additional capabilities to accustomed to the hypoxic leptomeningeal compartment.

All of the data discussed above are consistent with LM having a more malignant biological behavior. The more malignant behavior, the more widespread the metastases, and the poorer the outcome^26^. This got support from the observation of the shortened survival of LM (Fig. 5). The underlying mechanisms leading to the development of LM are still unclear, so additional research is needed.

We tried to construct nomograms to predict the development of LM or PM. The LM model had good predictive accuracy. Younger age, EGFR L858R, no pleural metastases, adrenal gland metastases and elevated CEA were risk factors. Of notice, some factors were referenced in other studies as well, which might suggest the reliability of our model^17, 18, 20, 21^. Our model was a comprehensive one, including factors from clinical (age and metastases) to laboratory findings (EGFR mutation and tumor markers). However, our model for PM consisting similar factors works poorly. These results indicated that the nomogram performed well in predicting LM, but not good enough for PM. The different capability of the models might be a reflection of the heterogeneity beneath the onset of PM, not easily covered by a few factors.

The major limitations of this study were the small size of the cohort and the lack of a prospective validation of the models. In addition, the data were retrospective with inherent selection bias. Thus, the conclusion of our results should be interpreted with caution, and prospective large sample-sized studies were urgently needed.

In conclusion, we found PM and LM, both considered as to BM, had different clinical characteristics and prognosis. Moreover, we constructed a simple nomograms model to predict the onset of LM in patients with advanced NSCLC. Our results might be important for further understanding of the brain metastasis and exploration of therapeutic targets.

## Data Availability

The datasets generated during and/or analysed during the current study are available from the corresponding author on reasonable request.
